# When to perform bone scintigraphy in patients with newly diagnosed prostate cancer? a retrospective study

**DOI:** 10.1186/s12894-017-0229-z

**Published:** 2017-06-12

**Authors:** Yiwei Lin, Qiqi Mao, Bin Chen, Liujiang Wang, Ben Liu, Xiangyi Zheng, Liping Xie

**Affiliations:** Department of Urology, The First Affiliated Hospital, School of Medicine, Zhejiang University, Qingchun Road 79, Hangzhou, 310003 Zhejiang Province China

**Keywords:** Prostate cancer, Prostate specific antigen, Gleason score, Bone scintigraphy

## Abstract

**Background:**

To determine when a bone scintigraphy investigation is appropriate in patients with newly diagnosed prostate cancer (PCa).

**Methods:**

We retrospectively reviewed 703 newly diagnosed PCa patients who were referred for bone scintigraphy. The association between age, prostate specific antigen (PSA), Gleason score (GS) and bone scintigraphy result were investigated by series of crude or stratified analysis.

**Results:**

Overall, 15.08% (106/703) patients had bone metastases. PSA and GS between positive bone scan group and negative bone scan group were significantly different, while age was not. The incidence of bone metastasis in patient with PSA < 20 ng/ml or GS < 8 was less than 10%, but increased dramatically with rising PSA and upgrading GS. In multivariate analysis, PSA ≥ 20 ng/ml (OR = 5.10, 95%CI (2.12-12.27)) and GS ≥ 8 (OR = 3.61, 95%CI (1.55-8.41)) were independently predictive of positive bone scan.

**Conclusions:**

Patients with PSA ≥ 20 ng/ml or GS ≥ 8 were in higher risk of bone metastasis, bone scintigraphy was recommended. But a bone scintigraphy is of limited value in PCa patients with PSA ≤ 20 ng/ml and GS ≤ 7.

## Background

Prostate cancer (PCa) is one of the leading malignant diseases in male in Western countries but it is relatively uncommon in China. However, recent widespread use of serum prostate-specific antigen (PSA) made PCa a growing health problem in China, with a bulky increase in incidence [1]. Early detection of bone metastases will be of great importance, as it can alert the clinician to the possible complications inherent in skeletetal destruction and reduce morbidity. Bone scintigraphy remains the most sensitive modality for detection of bone metastases, being superior to clinical evaluation, bone radiographs, serum alkaline phospatase measurement and prostatic acid phosphatase (PAP) determination [2].

However, routine use of bone scintigraphy staging is controversial. Subgroups of PCa patients with minimal risk for bone metastases can be safely excluded from bone scintigraphy. Numerous studies have demonstrated serum PSA, Gleason score (GS) and clinical tumor stage can be successfully used as indicators to predict which patients required bone scintigraphy for bone metastasis. According to a novel risk stratification tool proposed by Briganti et al.[3], bone scintigraphy might be considered only for patients with a biopsy GS > 7 or with PSA > 10 ng/ml and cT2/T3 disease prior to treatment. Ritenour et al. [4] recommend using GS as primary threshold to enhance predictability of positive bone scan in newly diagnosed patient with PCa. Bone scans should be done for patients with GS ≤ 7 when PSA ≥ 30 ng/ml, and for patients with GS > 7 when PSA ≥ 10 ng/ml. However, the criteria varied from different regions. Zaman et al. [5] found that there was an overall increased incidence of bone metastasis in newly diagnosed patients with PCa and even at PSA ≤20 ng/ml and GS ≤ 7 in Asian males, while other studies [6–8] indicated that bone scan can be eliminated for PCa patients with PSA < 10 ng/ml. Currently, limited data [9, 10] was available to strengthen which recommendation was more suitable for Chinese patients. Thus, we conducted a retrospective study in order to determine the diagnostic correlation among PSA, GS, clinical tumor stage and bone metastasis in newly diagnosed PCa patients in local population in China.

## Methods

The current study was approved by ethics committee at our hospital. It included newly diagnosed PCa patients from January 2011 to December 2014 in the Department of Urology at the First Affiliated Hospital of Zhejiang University in China. The consecutive 703 records of the patients were reviewed retrospectively for bone scan images, PSA levels and GS in biopsy, after excluding those with prior 5-alpha reductase inhibitors medication or surgical treatment for benign prostatic hyperplasia. All the bone scans were carried out as a routine clinical evaluation to further stage the prostate cancer prior to prostatectomy, radiotherapy or androgen deprivation therapy. The PSA tests were within 30 days of bone scan, and the most recent bone scan results after prostate biopsy were included in the study to avoid bias caused by delayed diagnosis of bone metastases during tumor progession.

The primary outcome measured was the presence of bone metastasis on bone scintigraphy. Whole body bone scintigraphy was performed using Tc-99-methylenediphosphonate (Tc 99 m MDP) and reviewed by 2 certified nuclear medicine physicians with extensive experience. The skeletal metastasis on bone scintigraphy was defined as either solitary or multiple asymmetric areas of increased tracer uptake presence, excluding tracer accumulations related to previous trauma and degenerative bone disease [11]. Patients equivocal bone scan findings (298 cases) also underwent computed tomography (98 cases), magnetic resonance imaging (MRI) (198 cases) and/or subsequent bone scan (2 cases) to confirm the bone scintigraphy findings. Conflicting evaluations were resolved by discussion. When needed, the consult from senior physician was sought.

The association between age, PSA, GS and bone scintigraphy result were investigated by series of crude or stratified analysis. Chi-square test was used for the comparison of proportions. Multivariate logistic regression analysis was used to compare the association between the independent variables (PSA and GS) and bone scan findings. To counter the skewness of the PSA values, a natural logarithm of PSA, lnPSA was used to generate a receiver operating characteristic (ROC) curve. All statistical analyses were performed using STATA, version 11.0 (StataCorp LP, College Station, TX, USA) and the outcome was considered significant only when the *p* value <0.05.

## Results

We identified 703 men who had been diagnosed with PCa and fulfilled our inclusion criteria. The mean age of included 703 patients was 69.9 years. The biopsy GS ranged from 6 to 10 with a mean value of 7.4, and the median PSA value was 22.1 ng/ml (interquartile range, 55.8 ng/ml). The proportion of patients with highly suspicious findings of bone metastases based on bone scan in our cohort was 15.08% (106/703). The comparative baseline characteristics of the two groups with different bone metastasis status were listed in Table 1. In the two groups, no difference between age was detected (for mean age, *p* = 0.13; for stratified subgroups, *p* = 0.96). However, there was a significant difference in PSA and GS between two groups (*p* <0.05).

**Table 1:** Patients’ baseline characteristics, *N* = 703

	All Patients	without bone metastasis (%)	with bone metastasis (%)	*p* value
Age, yr				
≤ 60	87	73 (12.2)	14 (13.2)	0.96
60-70	255	217 (36.3)	38 (35.8)	
≥ 70	361	307 (51.4)	54 (50.9)	
mean	69.7	69.9	68.7	0.13
PSA, ng/ml				
≤ 10	150	144 (24.1)	6 (5.7)	<0.05
10-20	184	175 (29.3)	9 (8.5)	
≥ 20	369	278 (46.6)	91 (85.8)	
mean	110.4	48.5	361.7	<0.05
GS				
< 7	132	125 (20.9)	7 (6.6)	<0.05
= 7	306	281 (47.1)	25 (23.6)	
> 7	265	191 (32.0)	74 (69.8)	
mean	7.5	7.3	7.9	<0.05

**Fig. 1:**
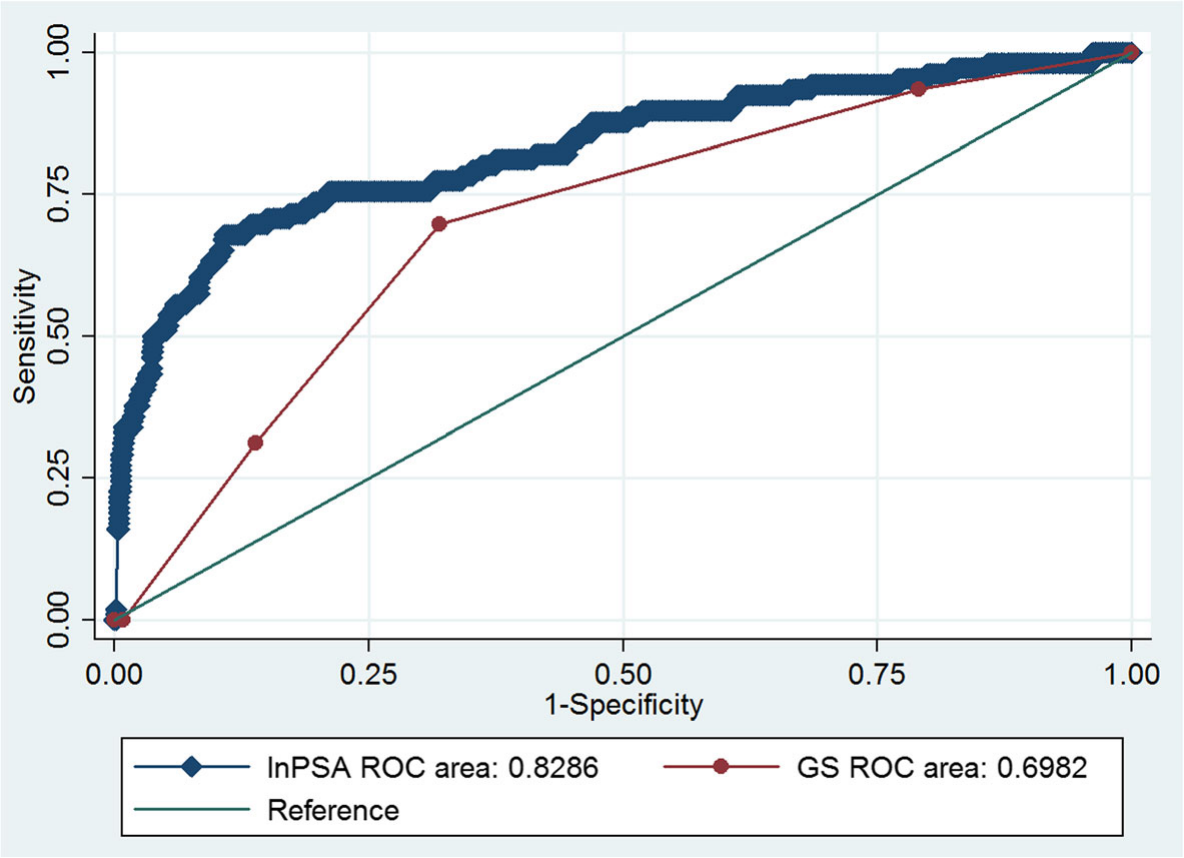
ROC curve analysis of lnPSA and GS for predicting bone metastasis

**Fig. 2:**
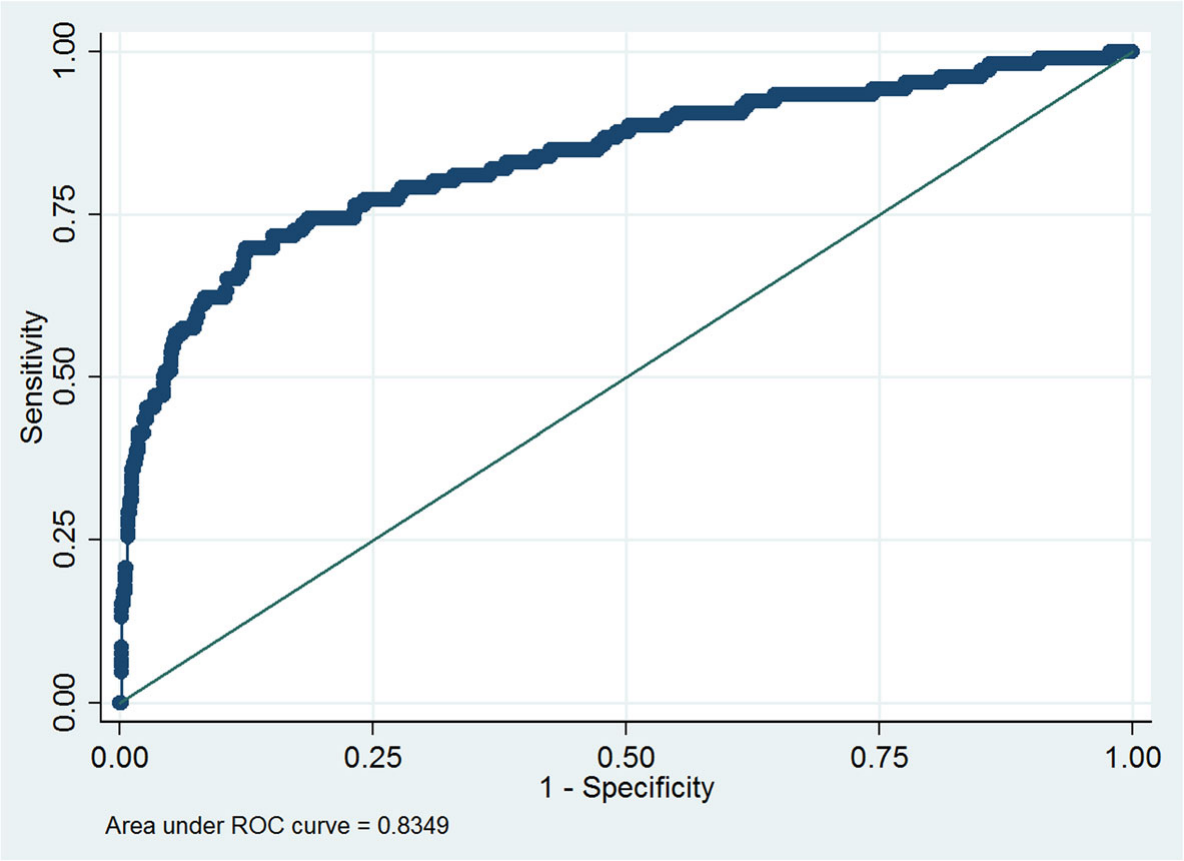
ROC curve analysis of combing lnPSA and GS for predicting bone metastasis

Moreover, we evulated the predictivity of PSA and GS on bone metastasis. Logistic regression with bony metastasis as the dependent variable and lnPSA and GS as independent variables was carried out to determine whether PSA and Gleason scores were additive. Both ln PSA and GS were statistically significant predictors of bone metastasis. However, receiver operating characteristic (ROC) curve for accuracy of ln PSA on bone metastasis has an area under curve (AUC) 0.829, which was superior over GS (AUC = 0.698) (Fig. 1). The multivariable model combing lnPSA and GS presented with AUC of 0.835 (Fig. 2), which was non-significant comparing with lnPSA alone (*p* = 0.21). The sensitivity and specificity of PSA at a cut-off (88 ng/ml) with maximal Youden’s index was 67.9% and 89.1% respectively, while GS at a cut-off (≥8) was a little bit more sensitive (69.81%) and less specific (68.01%) for diagnosing bone metastasis.

We further stratified the PSA into 3 groups: ≤10 ng/ml, 10-20 ng/ml, and ≥20 ng/ml. The prevalence of bone metastases increased progressively with PSA level. Bone metastasis was detected in 6 (6/150, 4.00%) patients with PSA below 10 ng/ml, and 9 (9/184, 4.89%) with PSA between 10 and 20 ng/ml. For the patients with PSA above 20 ng/ml, there was a dramatic increase in bone metastasis proportion (91/369, 24.66%). Correspondingly, multivariate analysis revealed higher risk (OR = 5.10, 95%CI (2.12-12.27)) of bone metastasis in patient with PSA > 20 ng/ml (Table 2).

**Table 2:** Predictors of positivity of bone scintigraphy using multivariate analysis

	OR (95% CI)	*p* Value
PSA, ng/ml		
≤ 10	*Reference*	
10-20	1.11(0.38-3.22)	0.85
≥ 20	5.10(2.12-12.27)	<0.05
GS		
< 7	*Reference*	
= 7	1.15 (0.47-2.76)	0.78
> 7	3.61 (1.55-8.41)	<0.05

For GS, larger proportion of bone metastasis was presented in patients with higher GS. 5.30% (7/132), 8.17% (25/306) and 27.92% (74/265) patients were found with bone metastasis in GS < 7, =7 and >7 respectively (Table 1). In multivariate analysis, GS (>7) was proven to be independently predictive of positive bone scan (OR = 3.61, 95%CI (1.55-8.41)) (Table 2).

## Discussion

Numerous studies have been conducted to find out proper criteria for ruling out bone metastases in patients with low risk of bone metastasis. PSA and biopsy GS were the most common parameters for assessing the bone metastasis risk. Oesterling et al. [12] were the first to address the possibility of serum PSA levels being able to predict bone scan results. They concluded that omitting bone scan for PSA less than 10 ng/ml is safe. The studies in some region of Asia also demonstrated that in well differentiated tumor, 10 ng/ml was the optimal cut-off PSA value for reserving bone scintigraphy [7, 8, 13]. However, American Urological Association (AUA) and the European Association of Urology (EAU) recommended that bone scan could be omitted in asymptomatic patients with well-differentiated tumor with PSA less than 20 ng/ml. The variation in criteria for bone scan recommendation was because of tremendous difference in incidence and stage pattern in different region worldwide [14]. In Asia, more aggressive and poorly differentiated PCa was presented, since twice as many Asian men had a GS of 8 or greater, the worst stage at presentation while comparing with non-Asian men [15]. Therefore, Zaman et al. [5] suggested that we must be careful in adopting current guidelines.

In China, the incidence of PCa is rapidly increasing, ranking eighth among the most common cancers in the male population in 2008 [1]. Although the reported incidence and mortality of PCa was much lower in China compared to Western countries, the mortality-to-incidence rate ratio (MR/IR) of PCa in China was found to be much higher [16]. These data suggest that higher proportion of advanced disease at the time of diagnosis, and that patient had a shorter survival time thereafter. Thus, precise staging for newly diagnosed PCa patient is of great importance, especially for bone metastasis staging, which has a profound influence on prognosis. Currently, bone scintigraphy is still regarded as the gold standard for detecting skeletal metastases in patients with newly diagnosed PCa. However, there was ongoing debate that whether we should propose bone scintigraphy as a routine staging modality for all patients, since the overuse of bone scan will result in unnecessary patient anxiety, radiation exposure, time consumption, and significant financial burden for both patients and healthcare system. In China, a clear PSA-driven cancer stage migration was presented after the initialization of PSA screening in some regions and more early stage disease was presented [17]. Thus, we believed the criteria for omitting bone scan in newly diagnosed case should be validated for Chinese patients.

Several studies have been conducted to search for the optimal bone scan indications for Chinese PCa patients, but with heterogeneous results [9, 10, 18]. Our current study revealed a similar trend that high PSA (>20 ng/ml) or poorly differentiated (GS > 7) were associated with increased bone metastasis risk in newly diagnosed PCa patients. PSA and GS could predict bone metastasis. In our cohort, only 3.76% (10/266) bone metastatic disease were with PSA < 20 ng/ml and GS < 8, which was almost negligible. Even more, we identified that PSA alone was powerful enough to predict bone metastasis, since the multivariable model combing lnPSA and GS did not significantly enhance the predictivity of lnPSA. For the predictability of GS, one issue still should be addressed when interpreting our results. The biopsy GS can probably bias surgical specimens, since GS upgrading between biopsy and surgical pathological specimens occurs 30–50% [19]. The accurate prediction of final GS largely depends on prostate biopsy schemes [20] and prostate size [19]. Thus, we speculated that the predictability of GS was weaker than PSA.

Meanwhile, several other issues still should be addressed when interpreting our results. Firstly, the follow-up time is short, which would prevent delayed diagnosis of bone metastatases. Then, metastatic lesions detected by bone scan were not histologically confirmed, thus the inconsistence between bone scan and histology would biased our results.

## Conclusion

Based on our findings, patients with PSA ≥ 20 ng/ml or GS > 7 were in higher risk of bone metastasis, and bone scintigraphy was strongly recommended for these patients. For the patients with PSA < 20 ng/ml or GS < 8, bone scintigraphy is of limited value unless a curative treatment is contemplated. On the other hand, considering the presence of metastatic disease in these low risk patients, a more precise stratification tool should be established to safely eliminate bone scintigraphy.

## Abbreviations

CI: Confidence interval
GS: Gleason score
OR: Odds ratio
PCa: Prostate cancer
PSA: Prostate specific antigen
